# Boosting Caloric Performances of Ni-Co-Mn-Ti Shape Memory Alloy for Multi-Scenario Refrigeration by Spark Plasma Sintering

**DOI:** 10.3390/ma18204691

**Published:** 2025-10-13

**Authors:** Hongyuan Tang, Ziqi Guan, Yanze Wu, Zhenzhuang Li, Jiaqi Liu, Xing Lu

**Affiliations:** 1 School of Material Science and Engineering, Dalian Jiaotong University, Dalian 116028, Chinalu@djtu.edu.cn (X.L.); 2 Shenyang National Laboratory for Materials Science, Institute of Metal Research, Chinese Academy of Sciences, Shenyang 110016, China

**Keywords:** Ni-Co-Mn-Ti alloys, spark plasma sintering, mechanical properties, elastocaloric effect, barocaloric effect

## Abstract

In this study, Ni_37_Co_13_Mn_33.5+*x*_Ti_16.5–*x*_ alloys with different particle sizes (75–150 μm, 50–75 μm, 0–50 μm) were successfully fabricated using spark plasma sintering under different processing conditions. By adjusting the composition of alloy and particle size, a significant transformation entropy change and the generation of a suitable amount of second phases along the grain boundaries were achieved in the SPS Ni_37_Co_13_Mn_34.5_Ti_15.5_ alloy with a particle size range of 0–50 μm. The mechanical properties of this optimized alloy were excellent, exhibiting a compressive strength of 2005 MPa and a fracture strain of 27%. Furthermore, under a loading rate of 0.28 s^−1^, the alloy demonstrated an adiabatic temperature change of up to 34.2 K. In addition, the alloy also exhibited a barocaloric effect under low-pressure conditions, achieving a substantial entropy change of 16.1 J·kg^−1^·K^−1^ and an estimated adiabatic temperature change of 11.2 K under 100 MPa pressure. Through these results, SPS Ni_37_Co_13_Mn_34.5_Ti_15.5_ alloy is proved to be a potential candidate for solid-state refrigeration applications.

## 1. Introduction

Refrigeration has become an increasingly essential aspect of modern life [1]. However, conventional refrigeration technologies, such as vapor compression systems, have been identified as significant contributors to global warming. This environmental impact underscores the urgent need for the development of novel, eco-friendly refrigeration technologies [2]. In the past few years, solid-state refrigeration based on shape memory alloys (SMAs) exhibiting various caloric effects has drawn considerable attention and is recognized as a promising alternative to traditional refrigeration systems [3]. The caloric effect refers to the adiabatic temperature change (Δ*T_ad_*) and isothermal entropy change (Δ*S_iso_*) induced in a material under the influence of an external field. These changes are primarily driven by the latent heat exchange related to reversible martensitic transformation (MT) [4,5]. Several types of caloric effects have been identified, including the magnetocaloric effect (MCE) [6,7], barocaloric effect (BCE) [8], electrocaloric effect (ECE) [9], and elastocaloric effect (eCE) [10,11]. Among these, the eCE, triggered by uniaxial stress fields, has demonstrated remarkable efficiency and practical implementation feasibility. As a result, it is widely considered the most promising candidate for real-world refrigeration applications [2,12].

Recently, significant attention has been focused on developing novel multifunctional materials, particularly Ni-(Co)-Mn-Z (Z = Ga, In, Sn, Sb, Ti) Heusler-type SMAs, which undergo a first-order MT from austenite to martensite upon cooling [3,7,13,14]. Among these materials, all-d-metal Heusler-type Ni-(Co)-Mn-Ti SMAs have emerged as promising candidates for solid-state refrigeration applications due to their excellent eCE properties [15,16,17]. Despite their attractive caloric properties, arc-melted Ni-(Co)-Mn-Ti SMAs generally exhibit poor mechanical performance, limiting their practical application. Enhancing the preferred orientation of these alloys has been shown to improve both their caloric effects and mechanical properties. Consequently, advanced techniques such as single-crystal growth and directional solidification have been utilized to improve the mechanical properties of these materials [18,19,20]. Several studies have illustrated that Ni-Mn-based Heusler alloys prepared via these methods can achieve remarkable mechanical strength. For instance, a dendritic-like Ni_50_Mn_31.6_Ti_18.4_ single-crystal alloy has achieved a high compressive strength exceeding 800 MPa [21]. Similarly, directional solidification has been shown to produce alloys with notably improved mechanical properties. The directionally solidified (Ni_50_Mn_28_Fe_2.5_Ti_19.5_)_99.4_B_0.6_ alloy exhibited a remarkable compressive strength of 2734 MPa [22,23,24]. While both single-crystal growth and directional solidification effectively improve mechanical performance, these methods are often complex, time-consuming, and costly. Therefore, there is a pressing need to develop a rapid, cost-effective method for producing SMAs with improved mechanical properties, enabling their practical application in solid-state refrigeration systems. In this context, spark plasma sintering (SPS) has emerged as an efficient and economical technique for alloy preparation. SPS has demonstrated significant potential in enhancing the mechanical properties of alloys [25,26]. Notably, sintered Ni-Mn-In alloys produced via SPS have exhibited impressive mechanical properties, achieving a compressive strength of 1800 MPa and a fracture strain of 19.3% [27]. These performance levels substantially exceed those of their arc-melted counterparts. This highlights the strong potential of SPS as a viable method for producing high-performance SMAs suitable for practical refrigeration applications.

In this study, the MT behaviors, including transformation temperatures (*M_s_*, *M_f_*, *A_s_*, and *A_f_*), MT entropy change (Δ*S_tr_*), mechanical properties, eCE and BCE of Ni_37_Co_13_Mn_33.5+*x*_Ti_16.5–*x*_ (*x* = 0, 0.5, 1) alloys with different powder particle sizes prepared via SPS were systematically investigated. The compressive strength of the alloys with various compositions was assessed, revealing that the Ni_37_Co_13_Mn_34.5_Ti_15.5_ alloy sintered using 0–50 μm powder achieved an impressive compressive strength of 2005 MPa. Furthermore, a remarkable Δ*T_ad_* up to 34.2 K was achieved under a loading rate of 0.28 s^−1^. In addition to its outstanding eCE properties, this alloy demonstrated significant BCE performance under low-pressure. With increasing pressure, the MT temperature exhibited a gradual rise, with a temperature shift rate (d*T*/d*P*) of 0.042 K·MPa^−1^. The entropy change value of barocaloric (Δ*S_BCE_*) reached 16.1 J·kg^−1^·K^−1^ under 100 MPa pressure, confirming the alloy’s efficient BCE behavior at low-pressure. These findings highlight that the SPS Ni-(Co)-Mn-Ti alloy, optimized in terms of composition and preparation conditions, successfully integrates excellent functional performance with enhanced mechanical properties. Consequently, this alloy presents itself as a potential candidate for future solid-state refrigeration applications.

## 2. Experiments

**Figure 1:**
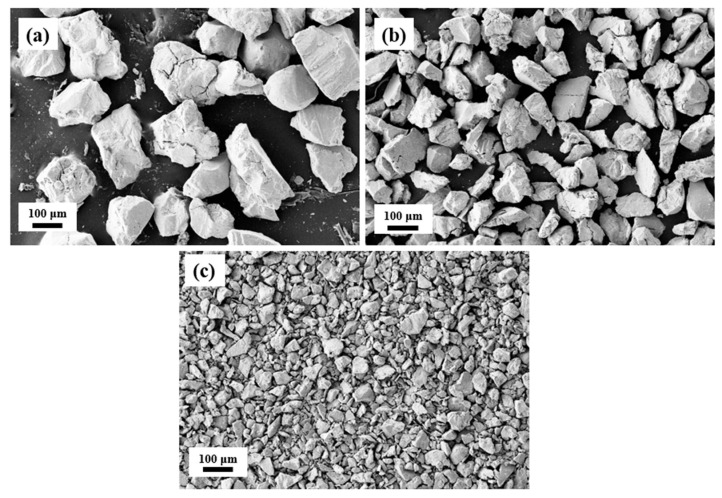
SEM pictures of the Ni_37_Co_13_Mn_34.5_Ti_15.5_ powder of different sizes. (**a**) 75–150 μm. (**b**) 50–75 μm. (**c**) 0–50 μm.

We initially prepared the Ni_37_Co_13_Mn_33.5+*x*_Ti_16.5–*x*_ (*x* = 0, 0.5, 1) alloys using conventional arc-melted. The arc-melted alloys were subsequently annealed at 1223 K for 48 h and quenched in water. The annealed samples were then mechanically ground into alloy powders, which were sieved into three distinct particle size ranges: 0–50 μm, 50–75 μm, and 75–150 μm, following the national standard sieve method. The micrographs illustrating the different powder sizes for the Ni_37_Co_13_Mn_34.5_Ti_15.5_ alloy are presented in Figure 1. The prepared powders were annealed at 873 K for 6 h before undergoing the sintering process. Sintering was conducted under a vacuum of 8 Pa with an applied pressure of 50 MPa at a temperature of 1223 K. The sintering duration varied among the samples, lasting 15, 20, 25, and 30 min, respectively. The same parameters were used to prepare Ni_37_Co_13_Mn_33.5_Ti_16.5_ alloy in our prior work [28], and this alloy exhibited excellent properties. Finally, the sintered samples were annealed once again at 1223 K for 24 h and subsequently quenched in water to enhance their properties.

A Differential Scanning Calorimetry Analyzer (DSC: TA-Q100, TA Instrument, Delaware, USA) was used to test the MT temperatures of each alloy using with a heating and cooling rate of 10 K·min^−1^. The specific heat capacity (*C_p_*) of Ni_37_Co_13_Mn_34.5_Ti_15.5_ alloy was also tested using this DSC analyzer with a heating rate of 2 K·min^−1^ from 300 K to 400 K. The mechanical properties of each alloy were measured on a universal mechanical testing machine (Shimadzu AG-Xplus/50 kN) equipped with a heating oven. The values of Δ*T_ad_* induced by external stress were measured at the temperature of *A_f_* +15 K by a K-type thermocouple clamped in the center of the sample surface. The microstructure of the alloys was observed by ZEISS SUPRA 55 scanning electron microscope (SEM, ZEISS, Baden-Württemberg, German), and imaging was performed using the secondary electron (SE) mode. The compositions of the alloys were determined by energy-dispersive spectroscopy (EDS), and the results averaged five different areas. Cylindrical samples (∅ = 3 mm, h = 300 μm) were cut from the SPS samples, then the samples were first ground to a thickness of 70–80 μm, followed by electrochemical polishing. These samples were prepared for the high-resolution transmission electron microscope (HRTEM), and it was utilized to determine the crystallographic characteristics of the martensite and austenite phases.

## 3. Results and Discussion

**Figure 2:**
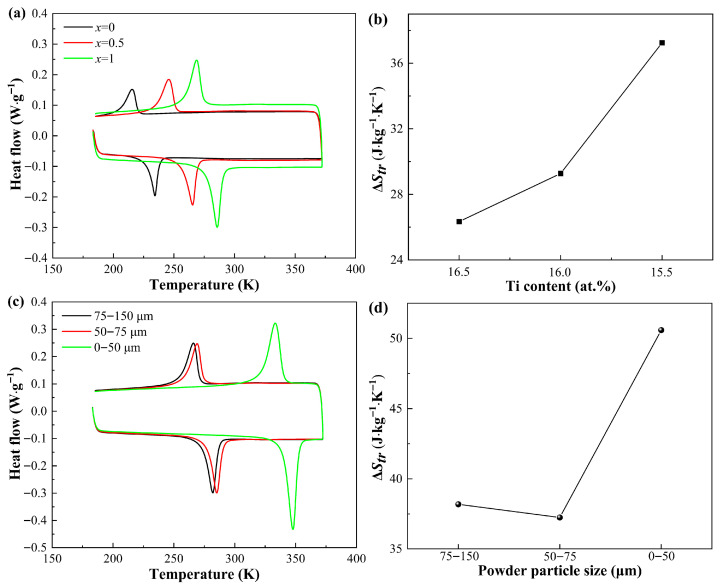
(**a**) DSC curves of alloys with varying compositions. (**b**) Entropy changes in alloys with varying compositions. (**c**) DSC curves of Ni_37_Co_13_Mn_34.5_Ti_15.5_ alloy sintered with powder of different particle sizes. (**d**) Entropy changes of Ni_37_Co_13_Mn_34.5_Ti_15.5_ alloy sintered with powder of different particle sizes.

Figure 2a displays the DSC curves of Ni_37_Co_13_Mn_33.5+*x*_Ti_16.5–*x*_ (*x* = 0, 0.5, 1) alloys sintered with 50–75 μm powder. It can be noted that as the Ti content decreases, the characteristic transformation temperatures progressively increase. This behavior can be attributed to the connection between the MT temperatures and the valence electron concentration (e/a) [29,30]. Generally, the MT temperatures tend to rise with increasing e/a values, which is directly influenced by the gradual substitution of Ti with Mn in the alloy composition [10]. Moreover, it is apparent that the characteristic transformation temperatures for all Ni_37_Co_13_Mn_33.5+*x*_Ti_16.5–*x*_ (*x* = 0, 0.5, 1) alloys are below room temperature (RT), indicating that these alloys predominantly exist in the austenitic phase at RT. Figure 2b shows the Δ*S_tr_* for the same set of alloys. It is noteworthy that the Δ*S_tr_* value increases progressively with the increase in Mn content. Among the examined compositions, the Ni_37_Co_13_Mn_34.5_Ti_15.5_ alloy exhibits the largest entropy change of 37.25 J·kg^−1^·K^−1^, making it a highly promising candidate for achieving an exceptional eCE near RT. Figure 2c illustrates the DSC curves of Ni_37_Co_13_Mn_34.5_Ti_15.5_ alloy sintered with powder of different particle sizes. The characteristic temperature rises with the reduction in the powder particle size. Figure 2d displays the Δ*S_tr_* of Ni_37_Co_13_Mn_34.5_Ti_15.5_ alloy with different particle size. It can be clearly observed that the Δ*S_tr_* value of 0–50 μm Ni_37_Co_13_Mn_34.5_Ti_15.5_ alloy is 50.59 J·kg^−1^·K^−1^, which is extremely higher than those of the other two alloys. This indicates that reducing the particle size during SPS can improve the Δ*S_tr_* of the alloy.

**Figure 3:**
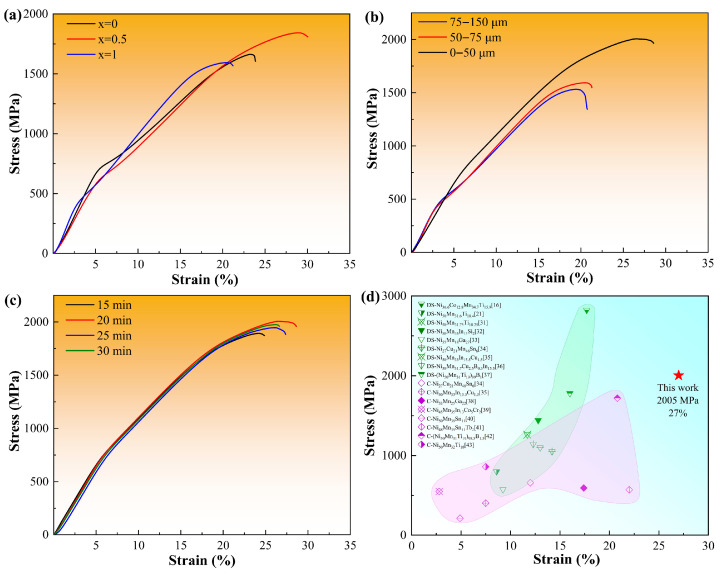
(**a**) Compressive stress–strain curves of samples with different compositions. (**b**) Compressive stress–strain curves of Ni_37_Co_13_Mn_33.5_Ti_15.5_ samples with different particle sizes. (**c**) Compressive stress–strain curves of Ni_37_Co_13_Mn_33.5_Ti_15.5_ samples with a particle size of 0–50 μm with different sintering times. (**d**) Comparison on the mechanical property for some SMAs prepared by different methods [16,21,31,32,33,34,35,36,37,38,39,40,41,42,43].

The eCE in SMAs originates from stress-induced martensitic transformation, making outstanding mechanical properties a crucial prerequisite for reaching remarkable elastocaloric performance. In this work, the compressive strength of the sintered Ni_37_Co_13_Mn_33.5+*x*_Ti_16.5–*x*_ (*x* = 0, 0.5, 1) alloys with different powder particle sizes and SPS times were carried out at RT. Figure 3a presents the stress–strain curves for the sintered Ni_37_Co_13_Mn_33.5+*x*_Ti_16.5–*x*_ (*x* = 0, 0.5, 1) alloys with a particle size range of 50–75 μm and an SPS duration of 20 min. The results indicate that these sintered alloys exhibit relatively high compressive strength, consistent with our previous findings [28]. Figure 3b shows the stress–strain curves for the sintered Ni_37_Co_13_Mn_34.5_Ti_15.5_ alloys with different particle sizes, where the SPS time was kept constant at 20 min. The results demonstrate that the compressive strength improves progressively as the particle size decreases. Notably, when the particle size is reduced to less than 50 μm, the compressive strength is measured to be 2005 MPa and the fracture strain reaches 27%. These values reflect significant enhancements of 30.9% (from 1532 MPa) and 42.1% (from 19%), respectively, compared to samples with particle sizes in the 75–150 μm range. Figure 3c illustrates the relationship between compressive strength and SPS time for the Ni_37_Co_13_Mn_34.5_Ti_15.5_ alloys with a particle size of 0–50 μm. The results reveal minimal variation in compressive strength with different SPS time, suggesting that once the composition and particle size are established, the SPS time has a negligible impact on the compressive strength. Moreover, as shown in Figure 3d, the compressive strength of the sintered Ni_37_Co_13_Mn_34.5_Ti_15.5_ alloy significantly exceeds that of most Ni-Mn-based Heusler SMAs obtained by as-cast or directional solidification, where DS represents directional solidified alloys, and C represents as-cast alloys. Furthermore, Table 1 lists preparation parameters and strength of some sintered alloy, it can be seen that the alloy prepared by the sintering method and parameters described in this study has a relatively high strength that exceeds most conventional sintered alloys. This highlights that SPS technology can effectively enhance the mechanical properties of Ni-Mn-Ti-based SMAs, thereby providing favorable conditions for achieving improved elastocaloric performance.

**Figure 4:**
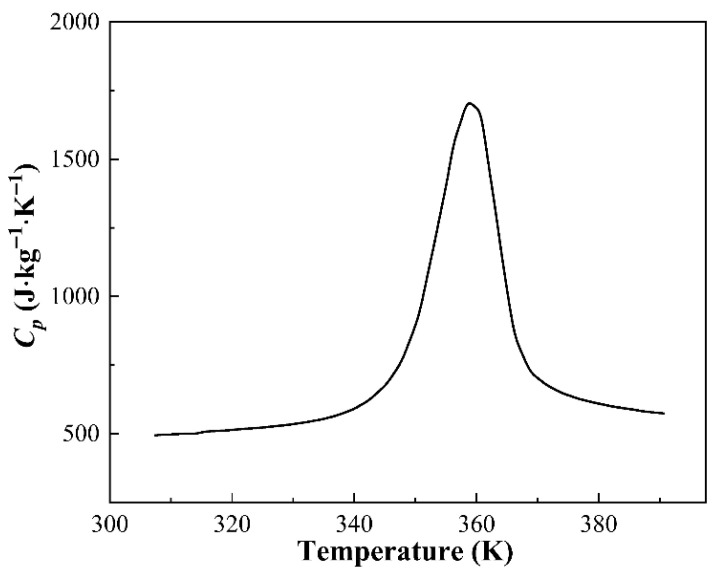
Specific heat capacity of Ni_37_Co_13_Mn_34.5_Ti_15.5_ alloy measured during heating.

To estimate the ideal Δ*T_ad_* for the eCE, the heat capacity *C_p_* in relation to temperature was measured, as shown in Figure 4. The ideal adiabatic temperature change without energy dissipation (Δ$T_{ad}^{ideal}$) for sintered Ni_37_Co_13_Mn_34.5_Ti_15.5_ alloy with the particle size of 0–50 μm and a sintering time of 20 min can be calculated by Δ$T_{ad}^{ideal}$ = (*T_0_*·Δ*S_tr_*)/*C_p_* [31], where *T_0_* is 346.2 K, Δ*S_tr_* is 50.59 J·kg^−1^·K^−1^ determined from Figure 2d, and *C_p_* is confirmed from Figure 4. Based on this calculation, the estimated Δ$T_{ad}^{ideal}$ for the sintered Ni_37_Co_13_Mn_34.5_Ti_15.5_ alloy is 35.7 K, demonstrating substantial potential for elastocaloric refrigeration applications. This result highlights the alloy’s ability to achieve significant temperature change under adiabatic conditions, further emphasizing its suitability as a promising alternative for solid-state refrigeration technology.

**Figure 5:**
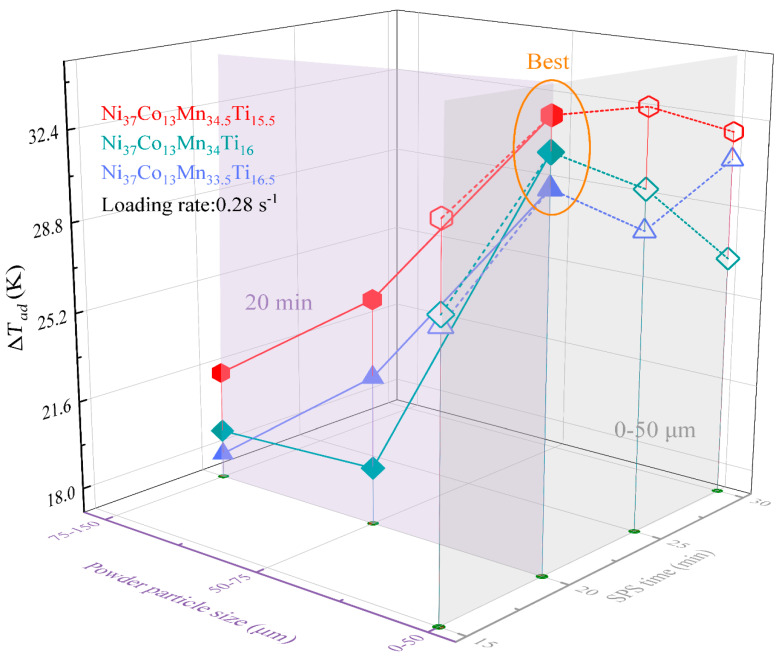
Elastocaloric Δ*T_ad_* values with different compositions, particle sizes and sintering time for Ni_37_Co_13_Mn_33.5+*x*_Ti_16.5–*x*_ (*x* = 0, 0.5, 1) alloys.

Figure 5 presents the measured Δ*T_ad_* values for SPS Ni_37_Co_13_Mn_33.5+*x*_Ti_16.5–*x*_ (*x* = 0, 0.5, 1) alloys under various conditions. The measurements were performed at a constant test temperature of *A_f_* +15 K, with samples subjected to a target strain of 15% at a loading rate of 0.28 s^−1^. The results reveal a clear trend: Δ*T_ad_* increases as the Ti content decreases when the powder particle size and SPS time remain constant. This enhancement is attributed to the gradual increase in Δ*S_tr_* as Mn gradually replaces Ti, as illustrated in Figure 2b. This is because the value of Δ*S_tr_* increases with the powder particle size becomes gradually smaller. Additionally, when composition and SPS time are fixed, Δ*T_ad_* shows a noticeable increase with decreasing particle size. Conversely, when composition and particle size are fixed, Δ*T_ad_* first rises and then declines with increasing SPS time. Remarkably, a colossal Δ*T_ad_* of 34.2 K was achieved in Ni_37_Co_13_Mn_34.5_Ti_15.5_ alloy under optimal conditions (0–50 μm particle size and 20 min SPS time), which corresponds to 95.8% of the ideal adiabatic temperature change Δ$T_{ad}^{ideal}$ (i.e., 35.7 K determined by Figure 4). Moreover, this outstanding Δ*T_ad_* value (i.e., 34.2 K) surpasses those reported for Heusler-type Ni-Mn-based SMAs fabricated by traditional arc-melted and directional solidification methods, as summarized in Table 2. These results highlight the significant potential of the SPS Ni_37_Co_13_Mn_34.5_Ti_15.5_ alloy as a promising replacement for high-performance solid-state refrigeration applications.

**Figure 6:**
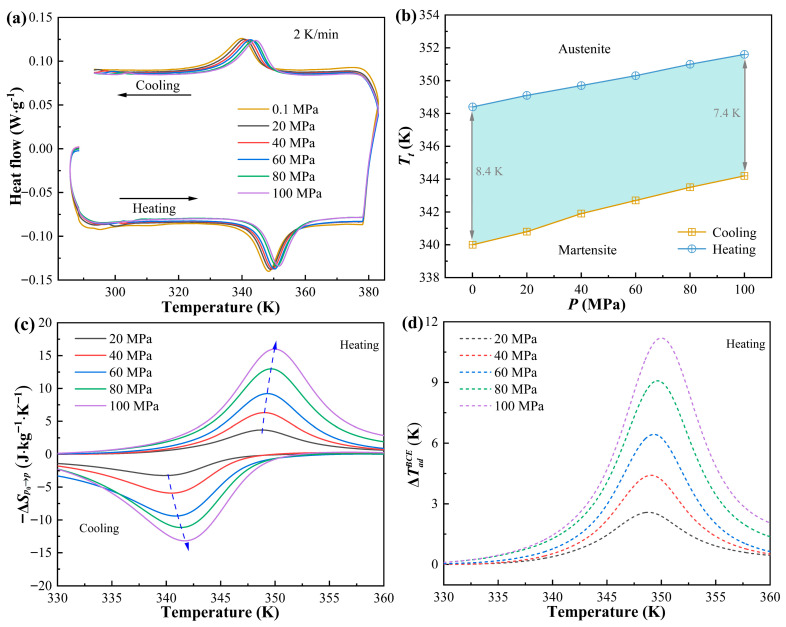
(**a**) DSC curves of Ni_37_Co_13_Mn_34.5_Ti_15.5_ alloy under different pressure. (**b**) A*_p_* and M*_p_* of Ni_37_Co_13_Mn_34.5_Ti_15.5_ alloy under different pressure. (**c**) Temperature dependence on Δ*S* under different pressure. (**d**) Temperature dependence on Δ$T_{ad}^{BCE}$ under different pressure.

The SPS Ni_37_Co_13_Mn_34.5_Ti_15.5_ alloy, which exhibits the most enhanced eCE, was further investigated for its BCE. The DSC curves of the samples, as illustrated in Figure 6a, reveal that the MT temperature increases with higher applied pressure. This behavior suggests that loading pressure is as effective as cooling in stabilizing the martensitic phase. From the results in Figure 6a, the peak temperatures of the forward (M*_p_*) and inverse (A*_p_*) MT shifts with pressure (d*T*/d*P*) for the 0–50 μm alloy were calculated to be 0.032 K·MPa^−1^ and 0.042 K·MPa^−1^, respectively. These values indicate that the present alloy’s A*_p_* and M*_p_* temperatures are relatively sensitive to hydrostatic pressure, signifying its potential to achieve a substantial BCE. Figure 6b shows the A*_p_* and M*_p_* of SPS Ni_37_Co_13_Mn_34.5_Ti_15.5_ alloy under varying pressures. The thermal hysteresis (A*_p_*−M*_p_*) declines from 8.4 K to 7.4 K as the pressure rises from 0 to 100 MPa. The reduction in thermal hysteresis is highly desirable as it can improves cycling stability and minimizes energy loss during repeated thermal cycles. The temperature dependence of the Δ*S_BCE_* and Δ$T_{ad}^{BCE}$ values reached 16.1 J·kg^−1^·K^−1^ and 11.2 K under 100 MPa pressure, respectively. These values are higher than some typical barocaloric metallic materials, including Ni_58.3_Mn_17.1_Ga_24.6_ alloy (i.e., 13.6 J·kg^−1^·K^−1^ under 1050 MPa) [63], Ni_44.6_Co_5.5_Mn_35.5_In_14.4_ alloy (i.e., 15.6 J·kg^−1^·K^−1^ under 598 MPa) [64], (MnNiGe)_0.91_-(FeCoGe)_0.09_ alloy (i.e., 5.2 K under 100 MPa) [65], and (MnCoGe)_0.96_-(CuCoSn)_0.04_ alloy (i.e., 3.4 K under 30 MPa) [66]. These findings demonstrate that the SPS Ni_37_Co_13_Mn_34.5_Ti_15.5_ alloy exhibits outstanding BCE performance under relatively low-pressure, highlighting its considerable potential for practical applications in solid-state refrigeration systems.

**Figure 7:**
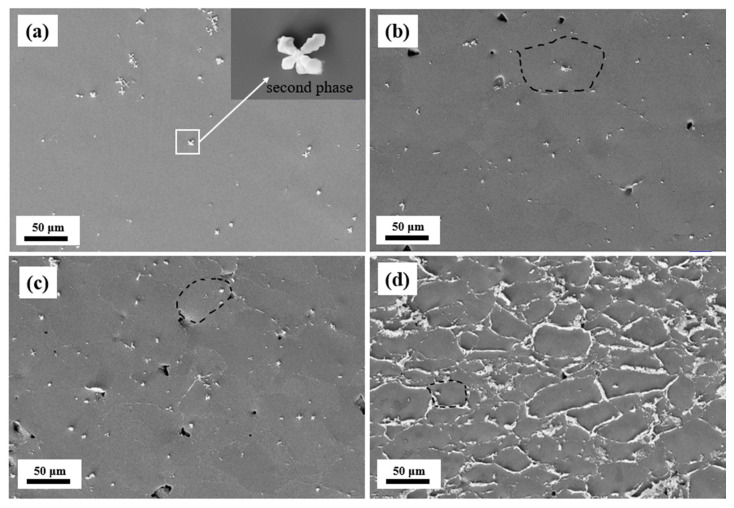
SEM pictures of the Ni_37_Co_13_Mn_34.5_Ti_15.5_ alloys. (**a**) The cast alloy. (**b**) 75–150 μm sintered alloy. (**c**) 50–75 μm sintered alloy. (**d**) 0–50 μm sintered alloy.

The above results clearly demonstrate that SPS technology is highly effective in strengthening the mechanical properties of SMAs. Building on this foundation, the SPS Ni_37_Co_13_Mn_34.5_Ti_15.5_ alloy exhibited remarkable eCE and BCE. To investigate the fundamental mechanism responsible for the exceptional properties of the sintered SMAs, a detailed microstructure analysis was conducted on both the arc-melted and SPS Ni_37_Co_13_Mn_34.5_Ti_15.5_ alloys with varying powder particle sizes (75–150 μm, 50–75 μm, and 0–50 μm), as shown in Figure 7. Figure 7b–d further illustrate that as the powder particle size decreases, the grain size (surrounded by black dashed lines) also decreases, leading to an increase in the number of grain boundaries. The increase in grain boundaries introduces greater resistance to dislocation movement under external stress, thereby enhancing the mechanical strength of the alloys [67]. Moreover, distinct precipitates existed in both the arc-melted and SPS alloys, which were predominantly located alongside the grain boundaries. In addition, the second phase inside the grain, as shown in the inset of Figure 7a, is Ti-rich second phase (Ti content: 78.7 at.%). Notably, in the SPS alloys, the amount of these precipitates increased progressively with decreasing powder particle size. This trend aligns with our previous observations in sintered Ni_37_Co_13_Mn_33.5_Ti_16.5_ alloys [28]. In general, the finer the grain size of an alloy, the higher its strength. Additionally, the segregation of secondary phases at grain boundaries can effectively prevent the alloy from intergranular fracture. The combination of reduced grain size and increased second-phase precipitates along grain boundaries is identified as the key factor contributing to the significant improvement of the mechanical properties in the SPS alloys. To further elucidate the composition of the matrix and the precipitates, EDS analysis (error bars: ±0.2%) was conducted, as presented in Table 3. The EDS results confirm that the matrix phase in the cast alloy aligns well with the nominal composition. Meanwhile, the precipitates observed in the SPS Ni_37_Co_13_Mn_34.5_Ti_15.5_ alloys were found to be rich in Ti and deficient in Mn and Ni, as displayed in Table 3. This suggests that the Ti element tends to segregate along the grain boundaries during the SPS process, forming a Ti-rich second phase that plays a critical role in strengthening the mechanical properties of the present alloy.

**Figure 8:**
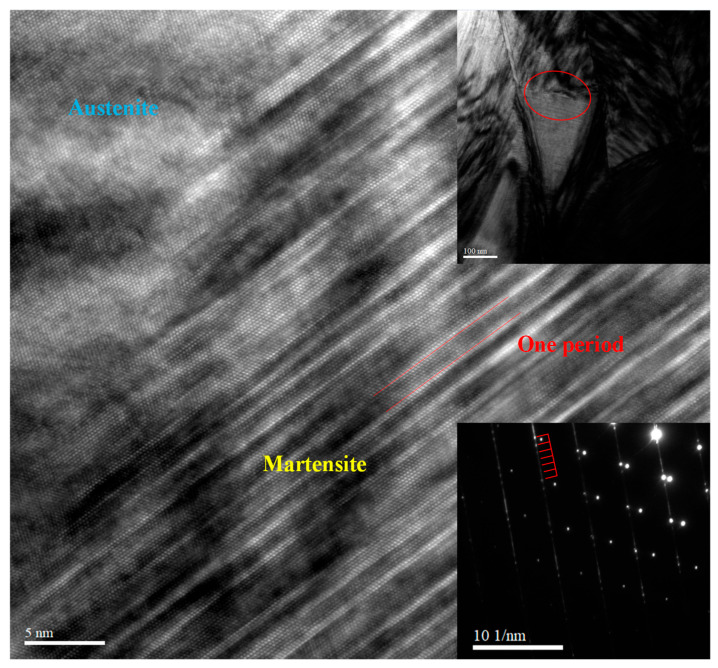
HRTEM lattice image for sintered Ni_37_Co_13_Mn_34.5_Ti_15.5_ alloy with particle size of 0–50 μm, taken at 340 K. The upper inset shows the matrix phases area image, and the lower inset shows the SAED pattern.

As previously demonstrated, the mechanical properties of the SPS Ni_37_Co_13_Mn_34.5_Ti_15.5_ alloy were significantly improved through grain refinement, achieved by reducing the powder particle size (Figure 7). To further investigate the presence of fine precipitates within the grains of the SPS Ni_37_Co_13_Mn_34.5_Ti_15.5_ alloy, HRTEM analysis was conducted. The HRTEM observations confirmed the existence of a small amount of fine precipitates within the grains (Figure 8 and the upper inset of Figure 8), which aligns with the SEM results (Figure 7). Furthermore, the microstructural examination revealed the coexistence of martensite and austenite phases within the selected region, as depicted in Figure 8. Through observing the selected area electron diffraction (SAED) pattern (the lower inset of Figure 8), it can be seen that there are five secondary diffraction spots between the two main diffraction spots, which is a typical feature of the martensite structure. This characteristics of such diffraction spots confirmed that the martensite phase adopts a six-layered modulated (6M) structure, while the austenite phase exhibits a cubic B2 structure. The presence of the 6M martensitic structure can enhance the homogenization of the atomic structure, which facilitates the occurrence of phase transformation, thereby improve the caloric effects of the alloy. These findings are consistent with previous reports [28,68], further validating the microstructural characteristics of the SPS Ni_37_Co_13_Mn_34.5_Ti_15.5_ alloy.

It is well known that the presence of an appropriate amount of second-phase precipitates distributed along grain boundaries can effectively enhance the mechanical properties of certain alloys [69]. In the present work, the observed increase in Ti-rich second-phase precipitates alongside the grain boundaries significantly contributes to strengthening grain boundary cohesion and impeding intergranular fracture [19]. This enhanced cohesion effectively inhibits crack initiation and propagation along grain boundaries, thereby making the overall mechanical properties of the alloy better. The enhancement in mechanical performance can be ascribed to two primary factors. Firstly, grain refinement increases the number of grain boundaries, which serves as an effective barrier to crack propagation. Secondly, the increased presence of Ti-rich second-phase precipitates along the grain boundaries further strengthens their cohesion, acting as a robust obstacle to crack formation and intergranular fracture. These combined effects provide a solid foundation for the remarkable mechanical properties observed in the SPS Ni_37_Co_13_Mn_34.5_Ti_15.5_ alloy.

## 4. Conclusions

In conclusion, the MT temperature, Δ*S_tr_*, mechanical properties, eCE, and BCE of the Ni_37_Co_13_Mn_33.5+*x*_Ti_16.5–*x*_ (*x* = 0, 0.5, 1) sintered alloys were comprehensively investigated. Notably, the sintered Ni_37_Co_13_Mn_34.5_Ti_15.5_ alloy with a particle size of 0–50 μm exhibited an impressive Δ*S_tr_* of 50.59 J·kg^−1^·K^−1^. The alloy also achieved exceptional mechanical properties, with a maximum compressive strength of 2005 MPa and a fracture strain of 27% at RT. Microstructural analysis via SEM and TEM revealed that the enhanced mechanical strength is due to the increased presence of Ti-rich second phases along the grain boundaries, which strengthen grain boundary cohesion and effectively impede intergranular fracture. Furthermore, a remarkable Δ*T_ad_* of 34.2 K was achieved under a high strain rate of 0.28 s^−1^, underscoring the alloy’s promising potential for practical elastocaloric refrigeration applications. In addition, the alloy demonstrated an outstanding barocaloric performance, achieving a great ideal Δ$T_{ad}^{BCE}$ of 11.2 K under a low pressure of 100 MPa. These results demonstrate that the SPS Ni_37_Co_13_Mn_34.5_Ti_15.5_ alloy successfully combines excellent mechanical properties with superior eCE and BCE performance, making it a highly potential candidate for efficient solid-state refrigeration applications in both high-pressure eCE and low-pressure BCE scenarios.

## Figures and Tables

**Table 1:** Preparation parameters and compressive/tensile strength of some sintered alloys.

Alloy	Sintering Method	Applied Pressure (MPa)	Sintering Time (Min)	Sintering Temperature (K)	Tensile/ Compressive Strength (MPa)	Ref.
Ni_37_Co_13_Mn_34.5_Ti_15.5_	SPS	50	20	1223	2005	This work
Ni_50_Mn_34.7_In_15.3_	SPS	50	15	1173	1800	[27]
Ta–10 wt%W	SPS	35	5	1873	693.41	[44]
Ni_43.75_Mn_37.5_In_12.5_Co_6.25_	SPS	50	10	1073	1440	[45]
40% Ni_49.8_Mn_28.5_Ga_21.7_/Cu	SPS	40	5	1073	865	[46]
Ti-46.5Al-2.15Cr-1.90Nb	SPS	50	7	1423	1820	[47]
Ni_45_Co_5_Mn_36.7_In_13.3_	SPS	40	5	1073	1900	[48]
Ni_48.8_Mn_29.7_Ga_21.5_	SPS	50	10	1173	1706	[49]
Ti-15Nb-25Zr-8Fe	SPS	50	10	1473	1920	[50]
93W-Ni-Fe	HIP	140	240	1573	1582.8	[51]
TI-6Al-2Sn-4Zr-2Mo	HIP	103	120	1123	1048	[52]

**Table 2:** Elastocaloric Δ*T_ad_* values of some caloric materials (DS represents the directional solidified alloy, and C represents the as-cast alloy).

Materials	Maximum Δ*T_ad_*	Conditions	Strain Rate	Refs.
Ni_37_Co_13_Mn_34.5_Ti_15.5_	34.2 K	loading	2.8 × 10^−1^	This work
DS-Ni_45.7_Co_4.2_Mn_37.3_Sb_12.8_	9.4 K	loading	1.8 × 10^−2^	[3]
DS-Ni_49_Mn_33_Ti_18_	33.6 K	loading	1.7	[22]
DS-Ni_55_Mn_18_Ga_26_Ti_1_	6.2 K	unloading	4 × 10^−2^	[33]
DS-(Ni_50_Mn_31_Ti_19_)_99_B_1_	17.8 K	loading	2.2 × 10^−2^	[37]
DS-Ni_50_Mn_30_Ti_20_	31.3 K	unloading	2.8	[53]
DS-Ni_55_Mn_18_Ga_27_	10.7 K	unloading	2.0 × 10^−1^	[54]
DS-Ni_50_Mn_31.5_Ti_18.5_	13.1 K	unloading	1.7	[55]
DS-Ni_50_Mn_33_In_14_Si_1_Cu_2_	18.2 K	unloading	1.0	[56]
DS-Ni_48.4_Mn_34.8_In_16.8_	4 K	unloading	1.7 × 10^−3^	[57]
DS-Ni_44_Mn_46_Sn_10_	18 K	unloading	2.8 × 10^−1^	[58]
C-Ni_35.5_Co_13.5_Mn_35_Ti_14.9_Gd_0.1_	13.5 K	loading	2.8 × 10^−2^	[10]
C-(Ni_50_Mn_31.5_Ti_18.5_)_99.8_B_0.2_	31.5 K	loading	5.33	[59]
C-(Ni_51.5_Mn_33_In_15.5_)_99.7_B_0.3_	6.6 K	loading	4.2 × 10^−2^	[60]
C-(Ni_51_Mn_33_In_14_Fe_2_)_99.4_B_0.6_	5.8 K	loading	2.8 × 10^−2^	[61]
C-Ni_43_Mn_41_Co_5_Sn_11_	9 K	unloading	3.4 × 10^−1^	[62]

**Table 3:** EDS results of the Ni_37_Co_13_Mn_34.5_Ti_15.5_ (at.%).

Preparation Methods	Phase	Ni	Co	Mn	Ti
Arc-melted	Matrix	37.2	13.2	34.1	15.5
SPS (75–150 μm)	Second phase (on the grain boundary)	25.2	9.0	25.6	40.2
SPS (50–75 μm)	Second phase (on the grain boundary)	25.0	8.8	24.2	42.0
SPS (0–50 μm)	Second phase (on the grain boundary)	27.2	9.1	25.7	38.0

## Data Availability

The original contributions presented in this study are included in the article. Further inquiries can be directed to the corresponding authors.

## References

[1] Zhao Z., Guo W., Zhang Z. (2022). Room-Temperature Colossal Elastocaloric effects in three-dimensional graphene architectures: An atomistic study. Adv. Funct. Mater..

[2] Panchenko E.Y., Yanushonite E.I., Eftifeeva A.S., Tokhmetova A.B., Kurlevskaya I.D., Tagiltsev A.I., Surikov N.S., Timofeeva E.E., Chumlyakov Y.I. (2022). Elastocaloric Effect in aged single crystals of Ni_54_Fe_19_Ga_27_ ferromagnetic shape memory alloy. Metals.

[3] Li Z., Li Z., Yang J., Li D., Yang B., Yan H., Nie Z., Hou L., Li X., Zhang Y. (2019). Large elastocaloric effect in a polycrystalline Ni45.7Co4.2Mn37.3Sb12.8 alloy with low transformation strain. Scr. Mater..

[4] Bonnot E., Romero R., Mañosa L., Vives E., Planes A. (2008). Elastocaloric Effect Associated with the Martensitic Transition in Shape-Memory Alloys. Phys. Rev. Lett..

[5] Wang Y., Liu C., Wang H., Li Z., Li J., Yang B., Yan H., Zuo L. (2024). Effect of burst-type martensitic transformation on superelastic and elastocaloric properties in a <116> oriented Cu_70.5_Al_17.5_Mn_12_ single crystal. Scr. Mater..

[6] Bez H.N., Pathak A.K., Biswas A., Zarkevich N., Balema V., Mudryk Y., Johnson D.D., Pecharsky V.K. (2019). Giant enhancement of the magnetocaloric response in Ni–Co–Mn–Ti by rapid solidification. Acta Mater..

[7] Yang J., Li Z., Zhang X., Yang B., Yan H., Cong D., Zhao X., Zuo L. (2023). Manipulation of thermal hysteresis and magnetocaloric effect in the Ni-Co-Mn-In alloys through lattice contraction: Effect of Ge substitution for In. Acta Mater..

[8] Kosugi Y., Goto M., Tan Z., Fujita A., Saito T., Kamiyama T., Chen W., Chuang Y., Sheu H., Kan D. (2021). Colossal Barocaloric Effect by Large Latent Heat Produced by First-Order Intersite-Charge-Transfer Transition. Adv. Funct. Mater..

[9] Mischenko A.S., Zhang Q., Scott J.F., Whatmore R.W., Mathur N.D. (2006). Giant electrocaloric effect in thin-film PbZr_0.95_Ti_0.05_O_3_. Science.

[10] Xu F., Zhu C., Wang J., Luo F., Zhu X., Xu J., Chen S., Wang J., Ma G., Chen F. (2023). Enhanced elastocaloric effect and mechanical properties of Gd-doped Ni-Co-Mn-Ti-Gd metamagnetic shape memory alloys. J. Alloys Compd..

[11] Xiang H.-Y., Guo Y.-X., Zhao X., Li Z., Yang B., Jia N., Yan H.-L., Zuo L. (2024). Large low-stress elastocaloric effect in Ti-Zr-Cr-Sn. Scr. Mater..

[12] Kim Y., Jo M.-G., Park J.-W., Park H.-K., Han H.N. (2018). Elastocaloric effect in polycrystalline Ni_50_Ti_45.3_V_4.7_ shape memory alloy. Scr. Mater..

[13] Li Z., Li Z., Li D., Yang J., Yang B., Wang D., Hou L., Li X., Zhang Y., Esling C. (2019). Influence of austenite ferromagnetism on the elastocaloric effect in a Ni_44.9_Co_4.9_Mn_36.9_In_13.3_ metamagnetic shape memory alloy. Appl. Phys. Lett..

[14] Zhang X., Li Z., Hu Y., Li J., Yang B., Yan H., Zuo L. (2024). Formation of a-b twin induced by tension in Ni-Mn-Ga magnetic shape memory alloys. Mater. Charact..

[15] Wei Z.Y., Liu E.K., Chen J.H., Li Y., Liu G.D., Luo H.Z., Xi X.K., Zhang H.W., Wang W.H., Wu G.H. (2015). Realization of multifunctional shape-memory ferromagnets in all-*d*-metal Heusler phases. Appl. Phys. Lett..

[16] Guan Z., Bai J., Sun S., Gu J., Liang X., Zhang Y., Esling C., Zhao X., Zuo L. (2022). Extraordinary mechanical properties and successive caloric effects with ultrahigh cyclic stability in directionally solidified Ni_36.6_Co_12.8_Mn_34.7_Ti_15.9_ alloy. Appl. Mater. Today.

[17] Zhao D., Liu J., Chen X., Sun W., Li Y., Zhang M., Shao Y., Zhang H., Yan A. (2017). Giant caloric effect of low-hysteresis metamagnetic shape memory alloys with exceptional cyclic functionality. Acta Mater..

[18] Karaca H. (2004). Compressive response of a single crystalline CoNiAl shape memory alloy. Scr. Mater..

[19] Guan Z., Bai J., Zhang Y., Gu J., Morley N., Zhang Y., Esling C., Zhao X., Zuo L. (2023). Ni-Co-Mn-Ti-B high performance multiferroic phase transformation material: Simultaneous modulation of mechanical properties and successive caloric effects by B doping. Mater. Today Phys..

[20] Wang Y., Liu C., Wang H., Li Z., Li J., Yang B., Yan H., Zuo L. (2023). Orientation dependence of elastocaloric effect in a Cu_71.3_Al_17.5_Mn_11.2_ single crystal. J. Alloys Compd..

[21] Li B., Li S., Yang B., Zhong H., Liu Z., Li D. (2023). Enhancing the elastocaloric effect and thermal cycling stability in dendritic-like Ni_50_Mn_31.6_Ti_18.4_ single crystal. J. Alloys Compd..

[22] Zhang G., Wang H., Li Z., Yang B., Yan H., Zhao X., Zuo L. (2023). Colossal elastocaloric effect in a A oriented Ni_49_Mn_33_Ti_18_ polycrystalline alloy. Scr. Mater..

[23] Guan Z., Bai J., Zhang Y., Gu J., Zhang Y., Esling C., Zhao X., Zuo L. (2023). Ultrahigh cyclic stability and giant elastocaloric effect in directionally solidified (Ni_50_Mn_28_Fe_2.5_Ti_19.5_)99.4B0.6 alloy. Scr. Mater..

[24] Li Z., Li Z., Li D., Yang J., Yang B., Hu Y., Wang D., Zhang Y., Esling C., Zhao X. (2020). Achieving a broad refrigeration temperature region through the combination of successive caloric effects in a multiferroic Ni_50_Mn_35_In_15_ alloy. Acta Mater..

[25] Fu Z., Chen W., Wen H., Zhang D., Chen Z., Zheng B., Zhou Y., Lavernia E.J. (2016). Microstructure and strengthening mechanisms in an FCC structured single-phase nanocrystalline Co_25_Ni_25_Fe_25_Al_7.5_Cu_17.5_ high-entropy alloy. Acta Mater..

[26] Tian X., Sui J., Zhang X., Zheng X., Cai W. (2012). Grain size effect on martensitic transformation, mechanical and magnetic properties of Ni–Mn–Ga alloy fabricated by spark plasma sintering. J. Alloys Compd..

[27] Kuang Y., Ai Z., Yang B., Hao X., Li Z., Yan H., Zhang Y., Esling C., Zhao X., Zuo L. (2020). Simultaneously achieved good mechanical properties and large magnetocaloric effect in spark plasma sintered Ni-Mn-In alloys. Intermetallics.

[28] Wu Y., Guan Z., Tang H., Li Z., Li Z., Wang Y., Lu X., Zuo L. (2025). Excellent mechanical properties and giant room-temperature elastocaloric effect in spark plasma sintered Ni-Co-Mn-Ti shape memory alloy. Phys. B Condens. Matter.

[29] Wu Y., Wang J., He Y., Wu H., Jiang C., Xu H. (2016). Microstructure and the correlated martensitic transformation of melt spinning Ni_50_Mn_29_Ga_21_−xTbx (x = 0–1) ribbons. Acta Mater..

[30] Tang X., Feng Y., Wang H., Wang P. (2019). Enhanced elastocaloric effect and cycle stability in B and Cu co-doping Ni-Mn-In polycrystals. Appl. Phys. Lett..

[31] Yan H.-L., Wang L.-D., Liu H.-X., Huang X.-M., Jia N., Li Z.-B., Yang B., Zhang Y.-D., Esling C., Zhao X. (2019). Giant elastocaloric effect and exceptional mechanical properties in an all-d-metal Ni–Mn–Ti alloy: Experimental and ab-initio studies. Mater. Des..

[32] Li Z., Li Z., Lu Y., Lu X., Zuo L. (2022). Enhanced elastocaloric effect and refrigeration properties in a Si-doped Ni-Mn-In shape memory alloy. J. Mater. Sci. Technol..

[33] Li D., Li Z., Zhang X., Yang B., Wang D., Zhao X., Zuo L. (2020). Enhanced cyclability of elastocaloric effect in a directionally solidified Ni_55_Mn_18_Ga_26_Ti_1_ alloy with low hysteresis. Scr. Mater..

[34] Yang J., Wang H., Li Z., Zou N., Yan H., Yang B., Zuo L. (2024). Crystallography of stress-induced martensitic transformation and giant elastocaloric effect in a A textured Ni_27_Cu_21_Mn_46_Sn_6_ shape memory alloy. Acta Mater..

[35] Wang H., Li D., Zhang G., Li Z., Yang B., Yan H., Cong D., Esling C., Zhao X., Zuo L. (2022). Highly sensitive elastocaloric response in a directionally solidified Ni50Mn_33_In_15.5_Cu_1.5_ alloy with strong A preferred orientation. Intermetallics.

[36] Huang X.-M., Zhao Y., Yan H.-L., Jia N., Tang S., Bai J., Yang B., Li Z., Zhang Y., Esling C. (2020). A multielement alloying strategy to improve elastocaloric and mechanical properties in Ni–Mn-based alloys via copper and boron. Scr. Mater..

[37] Wang H., Wang Y., Zhang G., Li Z., Yang J., Li J., Yang B., Yan H., Zuo L. (2024). Giant elastocaloric effect and improved cyclic stability in a directionally solidified (Ni_50_Mn_31_Ti_19_)_99_B_1_ alloy. Materials.

[38] Cong D., Wang S., Wang Y., Ren Y., Zuo L., Esling C. (2008). Martensitic and magnetic transformation in Ni–Mn–Ga–Co ferromagnetic shape memory alloys. Mater. Sci. Eng. A.

[39] Shen A., Sun W., Zhao D., Liu J. (2018). Influence of Cr on microstructure and elastocaloric effect in Ni–Mn–In–Co–Cr polycrystalline alloys. Phys. Lett. A.

[40] Tan C., Tai Z., Zhang K., Tian X., Cai W. (2017). Simultaneous enhancement of magnetic and mechanical properties in Ni-Mn-Sn alloy by Fe doping. Sci. Rep..

[41] Tian X., Zhang K., Tan C., Guo E. (2018). Influence of Doping Tb on the Mechanical Properties and Martensitic Transformation of Ni-Mn-Sn Magnetic Shape Memory Alloys. Crystals.

[42] Zhang G., Wang H., Li Z., Yang B., Yan H., Zuo L. (2024). Quasi-linear superelasticity and associated elastocaloric effect in boron-doped polycrystalline Ni-Mn-Ti alloys. Acta Mater..

[43] Wei Z.Y., Sun W., Shen Q., Shen Y., Zhang Y.F., Liu E.K., Liu J. (2019). Elastocaloric effect of all-*d*-metal Heusler NiMnTi(Co) magnetic shape memory alloys by digital image correlation and infrared thermography. Appl. Phys. Lett..

[44] Yu D., Bi X., Xing L., Zhang Q. (2023). Microstructural Evolution and Mechanical Properties of Spark Plasma Sintering of Tantalum-Tungsten Alloy. Metals.

[45] Bai J., Liu D., Gu J., Jiang X., Liang X., Guan Z., Zhang Y., Esling C., Zhao X., Zuo L. (2021). Excellent mechanical properties and large magnetocaloric effect of spark plasma sintered Ni-Mn-In-Co alloy. J. Mater. Sci. Technol..

[46] Gao P., Liu Z.-X., Tian B., Tong Y.-X., Chen F., Li L. (2022). Microstructure, phase transformation and mechanical properties of NiMnGa particles/Cu composites fabricated by SPS. Trans. Nonferrous Met. Soc. China.

[47] Wang D., Yuan H., Qiang J. (2017). The Microstructure Evolution, Mechanical Properties and Densification Mechanism of TiAl-Based Alloys Prepared by Spark Plasma Sintering. Metals.

[48] Tian B., Ren D.C., Tong Y.X., Chen F., Li L., Zheng Y.F. (2015). Microstructure, phase transformation and mechanical property of Ni-Co-Mn-In alloy prepared by spark plasma sintering. Mater. Sci. Forum.

[49] Tian X., Sui J., Zhang X., Feng X., Cai W. (2011). Martensitic transformation, mechanical property and magnetic-field-induced strain of Ni–Mn–Ga alloy fabricated by spark plasma sintering. J. Alloys Compd..

[50] Li Q., Sun H., Li J., Yuan X., Nakai M., Niinomi M., Nakano T. (2021). Influence of Sintering Temperature on Mechanical Properties of Ti-Nb-Zr-Fe Alloys Prepared by Spark Plasma Sintering. J. Mater. Eng. Perform..

[51] Hu B., Cai G. (2022). Effect of Hot Isostatic Pressing Process Parameters on Properties and Fracture Behavior of Tungsten Alloy Powders and Sintered Bars. Materials.

[52] Lopez M., Pickett C., Arrieta E., Murr L.E., Wicker R.B., Ahlfors M., Godfrey D., Medina F. (2020). Effects of Postprocess Hot Isostatic Pressing Treatments on the Mechanical Performance of EBM Fabricated TI^−6^Al^−2^Sn^−4^Zr^−2^Mo. Materials.

[53] Zhang G., Wang H., Li Z., Yang B., Yan H., Zuo L. (2023). Giant elastocaloric effect covering a wide temperature region in a directionally solidified Ni_50_Mn_30_Ti_20_ alloy. Scr. Mater..

[54] Li D., Li Z., Yang J., Li Z., Yang B., Yan H., Wang D., Hou L., Li X., Zhang Y. (2019). Large elastocaloric effect driven by stress-induced two-step structural transformation in a directionally solidified Ni_55_Mn_18_Ga_27_ alloy. Scr. Mater..

[55] Wang H., Li Z., Hou L., Li X., Yan H., Yang B., Zuo L. (2024). Large elastocaloric effect covering a broad temperature window in a composition-graded Ni_50_Mn_31.5_Ti_18.5_ alloy prepared by magnetic field-assisted directional solidification. Acta Mater..

[56] Li Z., Li Z., Lu Y., Lu X., Zuo L. (2022). Enhanced elastocaloric effect and specific adiabatic temperature variation in Ni-Mn-In-Si-Cu shape memory alloys. J. Alloys Compd..

[57] Huang Y., Hu Q., Bruno N., Chen J.-H., Karaman I., Ross J.H., Li J. (2015). Giant elastocaloric effect in directionally solidified Ni–Mn–In magnetic shape memory alloy. Scr. Mater..

[58] Zhang G., Li Z., Yang J., Yang B., Wang D., Zhang Y., Esling C., Hou L., Li X., Zhao X. (2020). Giant elastocaloric effect in a Mn-rich Ni_44_Mn_46_Sn_10_ directionally solidified alloy. Appl. Phys. Lett..

[59] Cong D., Xiong W., Planes A., Ren Y., Mañosa L., Cao P., Nie Z., Sun X., Yang Z., Hong X. (2019). Colossal Elastocaloric Effect in Ferroelastic Ni-Mn-Ti Alloys. Phys. Rev. Lett..

[60] Yang Z., Cong D., Sun X., Nie Z., Wang Y. (2017). Enhanced cyclability of elastocaloric effect in boron-microalloyed Ni-Mn-In magnetic shape memory alloys. Acta Mater..

[61] Yang Z., Cong D., Yuan Y., Wu Y., Nie Z., Li R., Wang Y. (2019). Ultrahigh cyclability of a large elastocaloric effect in multiferroic phase-transforming materials. Mater. Res. Lett..

[62] Cheng P., Zhang G., Li Z., Yang B., Zhang Z., Wang D., Du Y. (2022). Combining magnetocaloric and elastocaloric effects to achieve a broad refrigeration temperature region in Ni_43_Mn_41_Co_5_Sn_11_ alloy. J. Magn. Magn. Mater..

[63] He X.J., Xu K., Wei S.X., Zhang Y.L., Li Z., Jing C. (2017). Barocaloric effect associated with magneto-structural transformation studied by an effectively indirect method for the Ni_58.3_Mn_17.1_Ga_24.6_ Heusler alloy. J. Mater. Sci..

[64] He X., Wei S., Kang Y., Zhang Y., Cao Y., Xu K., Li Z., Jing C. (2018). Enhanced barocaloric effect produced by hydrostatic pressure-induced martensitic transformation for Ni_44.6_Co_5.5_Mn_35.5_In_14.4_ Heusler alloy. Scr. Mater..

[65] Kuang Y., Qi J., Xu H., Yang B., Li B., Li Z., Yan H., Zhang Y., Esling C., Zhao X. (2021). Low-pressure-induced large reversible barocaloric effect near room temperature in (MnNiGe)-(FeCoGe) alloys. Scr. Mater..

[66] Kuang Y., Hao X., Zhang Z., Yang B., Li B., Li Z., Yan H., Zhang Y., Esling C., Zhao X. (2022). Barocaloric and magnetocaloric effects in isostructurally alloyed (MnCoGe)-(CuCoSn) systems. J. Magn. Magn. Mater..

[67] Sun S., Bai J., Gu J., Guo K., Morley N., Gao Q., Zhang Y., Esling C., Zhao X., Zuo L. (2024). Extraordinary mechanical properties and room-temperature magnetocaloric effects in spark plasma sintered all-d-metal Ni-Co-Mn-Ti alloy. J. Alloys Compd..

[68] Beckmann B., Taubel A., Gottschall T., Pfeuffer L., Koch D., Staab F., Bruder E., Scheibel F., Skokov K.P., Gutfleisch O. (2025). Giant magnetocaloric effect of Ni-Co-Mn-Ti all-d Heusler alloys in high magnetic fields. Acta Mater..

[69] Zhou Z., Yang L., Li R., Li J., Hu Q., Li J. (2018). Martensite transformation, mechanical properties and shape memory effects of Ni-Mn-In-Mg shape memory alloys. Prog. Nat. Sci..

